# A Child–Parent Dyad Study on Adolescent Paranoia and the Influence of Adverse Life Events, Bullying, Parenting Stress, and Family Support

**DOI:** 10.1093/schbul/sbad119

**Published:** 2023-08-25

**Authors:** Jessica L Kingston, Lyn Ellett, Elizabeth C Thompson, Brandon A Gaudiano, Katarina Krkovic

**Affiliations:** Doctorate in Clinical Psychology, Department of Psychology, Royal Holloway, University of London, Surrey, UK; School of Psychology, University of Southampton, Southampton, UK; Department of Psychiatry and Human Behavior, Brown University, Providence, RI, USA; Department of Psychiatry and Human Behavior, Brown University, Providence, RI, USA; Department of Clinical Psychology and Psychotherapy, University of Hamburg, Hamburg, Germany

## Abstract

**Background:**

Paranoid beliefs commonly occur in the general adolescent population. Exposure to adverse life events (ALEs) and/or bullying are important environmental risk factors. The extent to which others, especially parents, are available to help a young person cope with stressful situations may offset this risk.

**Study Design:**

A cross-sectional adolescent-parent dyad design (*n* = 142 pairs) was used to test whether an adolescent’s perception of being supported by their family, and/or the parent’s perception of stress and burden in their parenting role, moderated the association between environmental risk and adolescent paranoid beliefs.

**Study results:**

Moderation analysis indicated that ALEs were significantly associated with adolescent paranoid beliefs when parents reported high stress and burden in their parenting role. Conversely, at low and moderate levels of parental stress, ALEs were unrelated to paranoid beliefs. Bullying was strongly associated with paranoia, with no moderation effects. The adolescent’s perception of support within their family had no moderating effects.

**Conclusions:**

Findings indicate that the focus of prevention should be shifted beyond just families of adolescents who are experiencing psychosis and/or have high “at-risk” profiles, to families of adolescents exposed to ALEs. Targeted support for parents to help reduce parental stress and burden, and help foster protective family environments even in the face of ALEs, is an important avenue for reducing the risk of paranoid beliefs in adolescents. Further research is required to better understand how to offset the deleterious effect of bullying on paranoid beliefs in adolescents.

## Introduction

Paranoia describes exaggerated beliefs that others intend to cause you harm,^1^ which exists on a continuum ranging from mild suspicion and mistrust to more distressing, persistent, and maladaptive beliefs (ie, persecutory delusions^2^). Adolescence is thought to be a critical period for the emergence of paranoid beliefs,^3^ with research showing that approximately 20% of general population UK adolescents experience paranoid beliefs on a weekly basis, which were associated with reduced self-esteem and well-being over a 6-week period.^4^ In adolescents seeking support for their mental health, the weekly prevalence of paranoid beliefs is substantially higher (35%), is significantly associated with clinician ratings of peer difficulties, depression and self-harm, and for those with persistent/increasing levels of paranoia, mental health problems were unremitting over a 3-month period.^5^ Paranoid beliefs are also one of the most commonly reported symptoms to co-occur with suicidality in teenagers.^6^ It is essential that research seeks to understand paranoid beliefs during this developmental period. During adolescence, beliefs about the self and others are under construction. Once consolidated, these beliefs are thought to have enduring effects across the lifespan.^7^ Interventions during the adolescent phase thus have great potential for supporting longer-term mental health.^8^ To date, however, the psychological understanding of paranoid beliefs in adolescents is limited, with no recognized conceptual framework to guide understanding and inform prevention and intervention strategies.

The persistence-proneness model proposes psychotic-like experiences (eg, paranoid beliefs, hallucinations) are common in young people and typically decline with age.^9^ However, exposure to environmental risk may disrupt attenuation. Adverse life events (ALEs) during childhood (ie, events that pose a threat to a child’s physical or psychological well-being) are one of the most widely replicated environmental risk factors for psychosis^10^ and are associated with paranoid beliefs in adolescents. For example, when interviewed, adolescents with elevated paranoia all reported experiencing threatening life event(s), with qualitative analysis suggesting that this may increase perceptions of feeling vulnerable in an unsafe world.^11^ In treatment-seeking children (8–14 years), frequency of stressful life events (eg, losses, danger to self and others) over the last 12 months were significantly associated with paranoid beliefs, even when controlling for age, gender, verbal ability, and hallucinations.^12^ Bullying (ie, repeated exposure to behavior from a peer that has the intention of causing harm and distress^13^) has gained particular attention. In treatment-seeking adolescents, the frequency of being bullied at school was significantly associated with paranoid beliefs, hallucinations, and dissociation in 14–16-year-olds.^14^ In a community sample of adolescents in the UK, more frequent and more severe reports of bullying (ranging from name calling to physical violence/threat) were associated with more distressing paranoid beliefs.^15^ Furthermore, cross-sectional mediation analysis suggested that bullying was associated with a tendency to overestimate threat in neutral social stimuli via increased paranoid thinking.^15^

Findings thus suggest that exposure to ALEs is associated with paranoid beliefs in adolescents. However, not all young people that experience ALEs develop paranoid beliefs. Indeed, the factors affecting this relationship are not well understood. The extent to which others, especially parents and teachers, are available to help a young person cope with stressful situations has been shown to play an important role on other mental health difficulties, such as anxiety.^16^ Likewise, in the bullying literature, a stable family environment and secure parent–child attachments can reduce the impact of bullying on later internalizing and externalizing problems.^17^ In adults, family emotional climate has been shown to play a central role in the trajectory of psychosis/schizophrenia, with high expressed emotion (eg, criticism, overinvolvement, and negative affective style) predicting early relapse, critical comments predicting risk of relapse, and warmth protecting patients from relapse.^18^ Parents separating from one another has also been associated with greater paranoid beliefs in Irish adolescents.^19^ Furthermore, in a national survey of over 10 000 adolescents from the USA, adolescents' views of their parents as being overprotective, indifferent, and abusive in their parenting role were significantly associated with the adolescents' self-reported paranoid beliefs.^20^ Existing research thus suggests that family environment, and in particular contexts characterized by high levels of warmth and low levels of expressed emotion, may help reduce the impact of ALEs on paranoid beliefs. However, existing literature on adolescent-parent relationships and paranoid beliefs is limited to one study,^20^ which only captured the views of the adolescent to assess family environment. Furthermore, the use of cross-sectional design precludes causal inference and other interpretations (ie, paranoia influences parental relationship and/or the perception of the parental relationship) cannot be ruled out.

Advancing existing research, this study used a dyad (adolescent-parent/carer) approach to investigate the impact of the family context on the association between ALEs and paranoid beliefs in adolescents. This design profits from simultaneously assessing perspectives from both members of the dyad, enabling us to assess the possible impact of parent factors on child factors. In the adolescent participants, we focused on the adolescent’s perception of being supported by their family. We hypothesized that for adolescents who feel supported by their family, and able to turn to their family for support in the context of recent ALEs, their threat response may be attenuated, and they may in turn be less likely to develop views about the world and others as hostile, unsafe, and threatening (ie, paranoid beliefs). We also focused on the parent’s perception of parenting their adolescent child. Here, we hypothesized that parental reports of low stress and burden in their parenting role would offset the vulnerability arising from ALEs. Our specific hypotheses were:

1. Adolescent exposure to environmental stress (ALEs and/or bullying in the last 12 months) will be significantly associated with elevated paranoid beliefs in adolescents.
2. Adolescent ratings of social support from their family (SSF, moderator A) and parents’ rating of stress and burden in their parenting role (parenting stress (PS), moderator B) will be significantly negatively associated with paranoid beliefs in the adolescent.
3. SSF and PS will moderate the strength of the association between environmental stress and adolescent paranoid beliefs. We predict that ALEs and bullying will be significantly positively associated with adolescent paranoid beliefs when adolescents report low (but not high) support from their family and when parents report high (but not low) parental stress.

## Methods

### Design

A cross-sectional dyad survey design was used. The hypothesized predictor was environmental stress (ALEs and bullying), the hypothesized dependent variable was adolescents' self-report of paranoid beliefs, and the hypothesized moderators were (1) adolescents’ perception of social support from their family (SSF) and (2) parents’ rating of stress and burden in their parenting role (PS).

### Participants

Qualtrics, an online participant recruitment service, was used to recruit adolescent-parent dyads from the UK. Three hundred and seventy-four participants qualified to take part in the study. Of these, 156 dyads completed the surveys. Fourteen were excluded for not meeting quality checks (final *n* = 142). Quota sampling was used to ensure a 50:50 gender split for adolescents completing the survey and a 50:50 split across the age groups of 14–15 and 16–17 years. This sample was powered (0.90) to detect a small-to-medium effect (*f*^*2*^  = 0.10) using linear multiple regression.

### Adolescent Measures

Means, standard deviations, and internal reliability values are reported in table 1.

**Table 1.** Correlations (Bootstrapped CIs) Between Hypothesized Predictors, Moderators, and Outcome Variable

				Correlations (Bootstrapped 95% CIs)			
	Alpha	Mean (*SD*)	Range	2	3	4	5
*Hypothesized outcome:*							
1. Ideas of Persecution	0.96	5.52 (8.67)	0–36	0.207* (0.008–0.387)	0.606** (0.427,0.746)	−0.121 (−0.260,0.044)	0.324** (0.145,0.472)
*Hypothesized predictors:*							
2. Adverse Life Events	—	1.19 (1.43)	0–6	—	0.307** (0.131,0.461)	−0.115 (−0.255,0.025)	0.186* (0.007,0.340)
3. Bullying	0.75	2.06 (2.89)	0–21		—	−0.099 (−0.233,0.057)	0.162* (−0.001,0.298)
*Hypothesized moderator:*							
4. Social Support Family	0.88	68.25 (12.1)	12–84			—	−0.327** (−0.530, −0.136)
5. Parental Stress^1^	0.86	38.73 (10.5)	19–66				—

*Note:* Only the Parental Stress Scale was completed by the parent.

*P < 0.05; ** *P* < 0.01.

#### Descriptive and Sociodemographic Variables.

Participants provided information on a range of sociodemographic variables. Those reported in this study included: Age, gender, household income, country of birth, and current diagnosis of a mental health disorder (yes/no).

#### The ALEs Scale.

Tiet et al^21^ is a 25-item self-report measure of negative life events (eg, someone in my family died, a close friend was seriously sick/injured) in which the adolescent indicates whether the event happened in the last 12 months (yes/no). For items rated “yes,” follow-up questions assess whether this was experienced as a good or bad experience (mostly good, mostly bad, NA, don’t know) and how affected they felt by the incident (not at all—a lot). Using established scoring procedures, a total adverse event score was computed by summing only those events that participants rated as “mostly bad” and as being affected “a little”, “some”, or “a lot”. Scores range from 0 to 25 and high scores indicate frequent ALEs. A random probability sample of 9–17-year-olds in the USA reported a mean frequency of 1.97 ALEs in the previous 12 months.^21^

#### The Brief Self-Report Measure of Adolescent Bullying – Victimization.

Murray et al^22^ is a 5-item measure of bullying in the last 12 months. Participants are presented with a brief introduction, followed by 5 examples of being bullied (eg, purposefully ignored; hit, bitten, and kicked). Adolescents estimate how many times over the last year (never, 1–2 times, 3–10 times, about once a month, about once a week, (almost) every day). Scores range from 0 to 25, with high scores indicating high rates of bullying.

#### The Revised Green et al, Paranoid Thoughts Scale.

Freeman et al^23^ is an 18-item measure with 2 subscales: Ideas of reference (8 items) and ideas of persecution (10 items). Items are rated on a 5-point scale (*0—not at all* to *4—totally*) and exhibit reliability across the paranoia continuum. To capture persecutory thoughts specifically, we used the ideas of persecution subscale. Scores range from 0 to 40, with high scores indicating high paranoid beliefs.

#### The Multidimensional Scale of Perceived Social Support.

Zimet et al^24^ is a 12-item scale assessing perceived social support in relation to significant others, family, and friends (1—very strongly disagree to 7—very strongly agree). Higher scores indicate greater perceived social support. Only the family subscale was used for this study. Scores range from 4 to 28, with high scores indicating high perceived family support.

### Parent Measures

#### The Parental Stress Scale.

Berry et al^25^ is an 18-item measure that assesses both positive (eg, finding enjoyment in parenting, feeling close to their child) and negative (eg, feeling overwhelmed by the role, finding a child’s behavior embarrassing, or stressful) aspects of being a parent. Items are rated from 1—strongly disagree to 5—strongly agree. Scores range from 18 to 90 with lower scores indicating low parental stress and high warmth.

### Procedure

Ethical approval was obtained from the host UK university. Potential participants were pre-registered adult Qualtrics users who registered as living with an adolescent child. Potential participants were contacted by Qualtrics to take part, and in all instances, consent from the parent was first obtained, after which their adolescent child was approached to take part. Only when both the parent and child consented to take part was access to the questionnaires granted. Consenting participants completed the questionnaires online via Qualtrics and were reimbursed for their time. To help prevent missing data, participants were required to respond to all questions on each page before progressing through the survey. To enhance the accuracy of the data, participants had to correctly respond to attention checks that were distributed through each survey (2 in the adolescent survey and 2 in the adult survey). Completion time was also monitored and those taking less than half of the median completion time were excluded. The mean completion time was 18 minutes for the adolescent survey and 17 for the adult survey. Participants with a geographical location that did not correspond with the stated location, and/or who did not consent to their data being used, and/or dropped out without completing all measures were excluded at source by Qualtrics. Participants not fulfilling quota conditions were also excluded.

### Statistical Analyses

Hypotheses 1 and 2 were tested using correlation analyses with bootstrapped confidence intervals. Hypothesis 3, predicting moderation, was tested using PROCESS^26^ macro for SPSS (model 1) with one outcome (adolescent paranoia), one predictor (ALEs or bullying), and one moderator variable (SSF or PS). Four moderation models were thus run in total. Predictors and moderators were centered around the sample mean. To account for heteroscedasticity issues, we used Cribari-Neto heteroscedasticity-consistent inference, as recommended by Hayes and Cai.^27^ Bootstrapping with 5000 bootstrap samples was utilized to account for normality issues. Moderation analyses were also computed controlling for family income and gender of the adolescent. Covariates did not alter findings. Statistics are therefore reported without covariates.

## Results

### Descriptives

Forty-four percent of adolescents and 24.6% of parents/carers were male. Mean ages were adolescents 15.4 years (*SD* = 1.09) and parents/carers 43.91 years (*SD* = 7.38). Ninety-one percent of the adolescents and 93.7% of parents identified as White British, 18.2% of adolescents and 33.8% of parents reported a current mental health condition confirmed by a doctor, with 10.6% adolescents and 23.2% of parents reporting that they currently take medication for that condition. Fifty-five percent of parents were married, 16.2% were single, 10.6% living with their partner, 3.5% were separated, 10% were divorced and 4.2% were widowed. The most commonly reported ALE was a member of the family dying (27.5%) and family member having an emotional/mental health problem (20%).

### Hypotheses 1 and 2

Table 1 shows that consistent with hypothesis 1, paranoid beliefs in adolescents were significantly associated with ALEs (small–medium ES^28^) and bullying (large ES). Consistent with hypothesis 2, adolescent paranoid beliefs were significantly associated with parental stress (medium ES), but counter to expectation, the young person’s perception of family social support was not significantly associated with their paranoid belief scores (small ES). Also consistent with expectation, lower levels of adolescent reported family social support was significantly negatively associated with higher levels of PS. Exposure to ALEs and bullying were also significantly positively correlated.

### Hypothesis 3

**Fig. 1.**
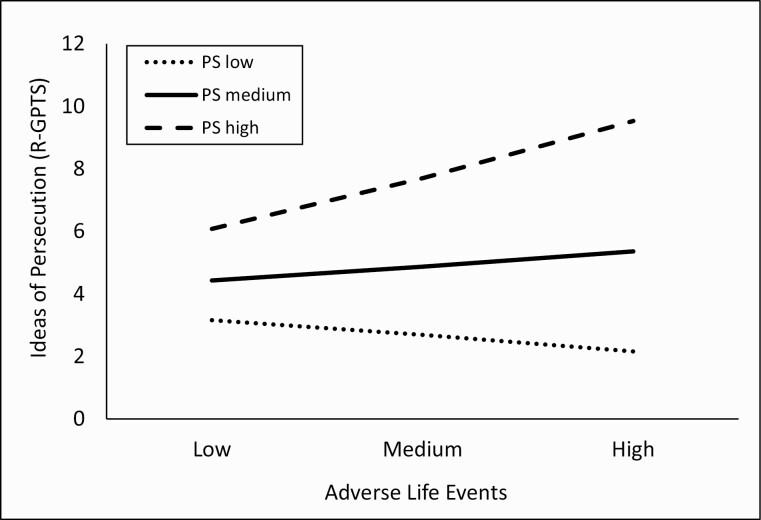
Line graph to show interaction between parental stress (parent rated) and adverse life events (adolescent rated) in predicting paranoid beliefs in adolescents. *Note:* PS, Parental Stress; RGPTS, Ideas of Persecution subscale of the Revised Green Paranoid Thoughts Scale. Only the Parental Stress Scale was completed by parents.

As reported in table 2, only one interaction term was significant, indicating that parental stress significantly modified the association between exposure to ALEs in the last 12 months and adolescent reports of paranoid beliefs in the last 2 weeks (model 2). The interaction accounted for 2.9% of variance (*R*^2^  = 0.029) in adolescent paranoia beliefs. The interaction (figure 1) showed that when the parent reports low and moderate levels of PS, exposure to ALEs was unrelated to adolescent paranoid beliefs. However, when parents reported high levels of PS, exposure to ALEs was strongly associated with paranoid beliefs in the adolescent.

**Table 2.** Moderation Analyses With Independent Variable (Adverse Life Events/Bullying), Moderator (Family Social Support and Parental Stress) and Outcome (Paranoid Beliefs)

	Coefficient	Standard Error	*t*	*P*	CI
*Model 1 R* = 0.237, *R*^2^ = 0.056, *F* = 2.308, *P* = .079					
Adverse life events	1.129	.580	1.945	.053	−0.019; 2.276
Social support family	−0.212	0.233	−0.908	.366	−0.673; 0.250
Interaction effect	−0.113	0.185	−0.611	.542	−0.479; 0.253
*Model 2 R* = 0.395, *R*^2^ = 0.156, *F *= 4.97, *P* = .003					
Adverse life events	0.558	0.506	1.102	.272	−0.443; 1.559
Parental stress	0.245	0.078	3.153	.002	0.091: 0.398
Interaction effect	0.095	0.046	2.070	.040	0.004; 0.186
*Model 3 R* = 0.620., *R*^2^ = 0.384, *F* = 14.76, *P* < .001					
Bullying	1.850	0.316	5.863	. < 0.001	1.226; 2.474
Social support family	−0.234	0.195	1.200	.234	−0.620; 0.153
Interaction effect	0.107	0.086	1.247	.214	−0.629; 0.278
*Model 4 R* = 0.648, *R*^2^ = 0.419, *F* = 11.64, *P* < .001					
Bullying	1.682	0.584	2.878	.005	0.526; 2.837
Parental stress	0.193	0.084	2.287	.024	0.026; 0.359
Interaction effect	0.005	0.092	0.055	.957	−0.177; 0.187

*Note:* The Parental Stress Scale was completed by parents. Co-varying for family income and adolescent gender does not alter significant findings.

Examining the adolescent’s perception of social support from their family (model 1), ALEs were significantly associated with paranoid beliefs, but family social support was not. In models with bullying as the independent variable, bullying had a significant main effect on adolescent paranoid beliefs in both moderation models. Accounting for the role of bullying, the adolescent's perception of family social support (model 3, table 2) was not significantly associated with paranoia; however, parental stress was (model 4, table 2). In model 4, both bullying and parental stress accounted for significant unique variance in paranoid beliefs, but no interaction was observed.

## Discussion

The current study used a dyad design to investigate whether adolescents’ perception of social support within their family and/or parental stress from the parents’ perspective moderated the impact of ALEs and/or bullying on paranoid beliefs in adolescents.

Paranoia had small correlations with ALE and perceived family support, a medium correlation with parent-reported stress, and a large correlation with bullying. Consistent with previous research,^29^ ALEs and bullying were also significantly correlated (medium ES). In model 2, parental stress significantly exacerbated the association between ALEs and paranoid beliefs, such that ALEs were only associated with paranoid beliefs when parents reported high stress and burden in their parenting role. This finding is consistent with literature on individuals at risk of, or experiencing, schizophrenia/psychosis where family environments high in criticism and overinvolvement, and low in warmth, play a pivotal role in determining persistence and relapse of psychotic symptoms.^18,30^ Similar findings have been reported for the impact of family functioning (ie, problem-solving, support, and communication).^31^ For example, Thompson et al^32^ reported that for adolescents at clinical high risk for psychosis, positive symptoms were associated with impaired social and role functioning when family functioning was low but not high. By focusing on a general population sample, our findings advance existing research by highlighting the importance of family environment in the emergence of paranoid beliefs at an early stage of development, in individuals who may not typically be identified as “at-risk.” Furthermore, our findings emphasise the potential scope for parents to be supported in actively offsetting the risk of paranoid beliefs developing in their adolescent children. Since parental stress was found to modulate the relationship between ALEs and paranoid beliefs, the focus of prevention should be shifted beyond just families of adolescents who are experiencing psychosis and/or have high “at-risk” profiles, to families of adolescents exposed to ALEs.

Consistent with previous research in clinical and nonclinical adult and adolescent samples, we found a large association between adolescents’ reports of being bullied and paranoid beliefs.^14,15,33^ However, although bullying and parental stress were both significantly associated with paranoid beliefs, no interaction was found. Similarly, in model 3, bullying was significantly associated with paranoid beliefs, but the adolescents’ perception of social support from their family was not, and no moderation effect was observed. Likewise, in model 1, the adolescents’ perception of social support from their families did not moderate the impact of ALEs on paranoia. These findings suggest that unlike in the case of ALEs, the association between being bullied and paranoid beliefs cannot readily be offset by a warm parenting context or by the adolescents’ perception of support from their family. Furthermore, the adolescents’ perception of family support in the context of ALEs was unrelated to paranoid beliefs.

The finding that adolescents’ perception of family support did not moderate the impact of ALE or bullying on paranoid beliefs adds to an already inconsistent literature on social support during adolescence. Although in some studies, social support at home and school has been found to offset the negative impact of bullying and ALEs on internalizing and externalizing problems^17^ and suicidal behavior,^34^ similar findings to ours have been reported for adolescent anxiety. For example, in a longitudinal study on trajectories of anxiety during adolescents, Spence et al^16^ reported that high levels of peer victimization prospectively predicted anxiety over time, which was not moderated by perceived social support from parents, peers, school, or a strong sense of belonging at school. Qualitative interviews with young people suggest that they often do not discuss paranoid beliefs with others^11^ and it is possible that even in families where adolescents feel cared for and supported, they may not disclose paranoid fears to family members. Adolescence is also a period of transition whereby systems of support outside of the family gain increasing significance. It is therefore possible that other sources of support, such as peers are worthy of investigation. Spence et al^16^ further raise the possibility that parents may not have the skills required to assist effectively when adolescent children are experiencing bullying and that high levels of support during this time may inadvertently undermine the adolescent’s independence and reinforce perceptions of the adolescent being vulnerable and unable to cope. An important difference between the 2 risk factors (ALEs and bullying) is that many of the ALEs either impacted the whole family (eg, family member dying, moving) or at least were events that parents would be aware of (eg, losing a friend). As such, parents may have been more attuned to the nature of the difficulty that their adolescent child was facing, giving more opportunity for them to play a role in buffering the ALE to paranoia link.

To the best of our knowledge, this is the first study to investigate the interaction of ALEs and family environment on adolescent paranoid beliefs, as well as being unique in obtaining the perspective of both the adolescent and parent. Furthermore, contrary to previous research that often relies on retrospective accounts from adult participants reporting on their childhood, in our study, measures were taken during this critical developmental phase. However, findings should be considered in light of some limitations. Although the gender and age distribution of adolescent participants was good, generalizability of the findings is hindered by most participants identifying as White British. Research on mild psychotic experiences in adolescents has identified the multifactorial nature of these experiences and the potential role of school ethnic density in understanding paranoia.^35^ Future research would benefit from closer examination of paranoia in adolescents from minoritized groups, which could benefit from focusing on specific forms of bullying such as racism and racialized bullying. Data were also self-report and, of particular note, there was a large correlation between the RGPTS and bullying (*r* = 0.606). It is possible that for some youth, what is conceptualized as “paranoia” on the RGPTS could actually be a reasonable interpretation of social slights or judgment (ie, beliefs due in part to bullying, and not paranoia, per se). Likewise, individuals with a tendency to perceive others as intending them harm may overestimate instances of bullying. Future research exploring a more descriptive and nuanced experience of paranoia, such as by using structured or qualitative interviews, informant (eg, parent or teacher) reports, or by assessing interpretations of ambiguous social scenarios,^36^ virtual reality, or in vivo social experiments to assess paranoia may help to overcome this issue. Relatedly, the RGPTS was developed in adults and validation in adolescents is only recently underway.^37^ Furthermore, the level of paranoia in this sample is low and replication in young people with clinically elevated scores is an important next step. Although there was temporality in the measurement of variables (ie, ALEs/bullying in the last 12 months, paranoid beliefs in the last 2 weeks), the design was nonetheless cross-sectional and cannot speak to the causal influence of independent and moderating variables on paranoia. Longitudinal prospective cohort studies, where ALEs, bullying, and paranoid beliefs can be tracked in real-time are an important next step, as well as examining whether these findings extend to other psychotic-like experiences (eg, hallucinations, delusions). Finally, the use of a composite measure of ALEs, although commonplace, privileges frequency over the nature of adversity. Future research would benefit from examining in more detail the nature of different types of adversities so as not to overlook specific adversities (as exemplified by the findings for bullying compared to ALEs).

Our findings clearly highlight that one important avenue for reducing the risk of paranoid beliefs in adolescents is via targeted support for parents, to help reduce parental stress and burden, and help foster protective family environments, even in the face of ALEs. This is especially the case given the association between family environment and risk for longer-term mental health difficulties identified in previous research.^19^ Findings also highlight the need for effective interventions to reduce the impact of bullying on paranoid beliefs. It is possible that paranoid beliefs are a largely under-acknowledged consequence of bullying that would benefit from being proactively anticipated and discussed with young people, so that fears can be put into context and prevented from becoming generalized. Greater understanding of paranoid beliefs amongst professionals in school and clinical settings is likely to be an important next step. Understanding the psychological mechanisms that link risk factors to paranoid beliefs during this developmental phase is also an important avenue for research. Negative beliefs about the self and others, and heightened negative affect, have reliably been identified as contributing to the development of paranoid beliefs in adults.^38^ As outlined by others,^18,39^ attachment relationships within the family are influential in shaping self and other beliefs, and a young person’s perception of themselves as vulnerable to harm. The role of attachment (to parents, teachers, and peers) and an adolescent’s evolving social identity^40^ are likely to be important in developing models of paranoid beliefs during adolescence. It is essential that future research uses longitudinal designs (to test causality), that it focuses on obtaining detailed accounts of adolescents' experiences of paranoia as they occur dynamically in daily life rather than relying exclusively on predefined questionnaires, and that research attends to culture and the potential need for different explanatory models by virtue of differences across cultures.^41^

In conclusion, ALEs were associated with elevated paranoid beliefs in adolescents only in the presence of high parental stress. Bullying, on the other hand, was uniquely associated with paranoia and neither the adolescent’s perception of family support nor the context of a warm and positive parenting approach offset this. Supporting parents who report high levels of stress (eg, via schools, communities), especially in the context of ALEs, may be an important avenue for reducing paranoia in adolescents.
